# Leflunomide Loaded Chitosan Nanoparticles for the Preparation of Aliphatic Polyester Based Skin Patches

**DOI:** 10.3390/polym13101539

**Published:** 2021-05-11

**Authors:** Stavroula G. Nanaki, Sophia Andrianidou, Panagiotis Barmpalexis, Evi Christodoulou, Dimitrios N. Bikiaris

**Affiliations:** 1 Laboratory of Polymer Chemistry and Technology, Department of Chemistry, Aristotle University of Thessaloniki, 54124 Thessaloniki, Greece; sgnanaki@chem.auth.gr (S.G.N.); sofia.andrianidou@gmail.com (S.A.); evicius@gmail.com (E.C.); 2 Department of Pharmaceutical Technology, School of Pharmacy, Aristotle University of Thessaloniki, 54124 Thessaloniki, Greece; pbarmp@pharm.auth.gr

**Keywords:** leflunomide, chitosan, aliphatic polyesters, poly(l-lactic acid), poly(lactic co glycolic acid), nanoparticles, skin patches, controlled release

## Abstract

In the present study, the preparation of controlled-released leflunomide (LFD)-loaded skin patches was evaluated, utilizing the combination of chitosan (CS) nanoparticles (NPs) incorporated into suitable poly(l-lactic acid) (PLLA) or poly(lactic-*co*-glycolic acid) (PLGA) polyester matrices. Initially, LFD-loaded CS NPs of ~600 nm and a smooth surface were prepared, while strong inter-molecular interactions between the drug and the CS were unraveled. In the following step, the prepared LFD-loaded CS NPs were incorporated into PLLA or PLGA, and thin-film patches were prepared via spin-coating. Analysis of the prepared films showed that the incorporation of the drug-loaded CS NPs resulted in a significant increase in the drug’s release rate and extent as compared to neat LFD-loaded polyester patches (i.e., prepared without the use of CS NPs). In-depth analysis of the prepared formulations showed that the amorphization of the drug within the matrix and the increased wetting properties of the prepared CS NPs were responsible for the improved thin-film patch characteristics.

## 1. Introduction

Leflunomide (LFD) is an orally administrated immunomodulator and anti-rheumatic drug used for the treatment of rheumatoid arthritis (RA) [1,2]. LFD is a prodrug that gets rapidly converted via an isoxazole ring cleavage into the active metabolite Teriflunomide (TFL) [3,4,5]. Its main action against RA is the inhibition of the de novo pyrimidine synthesis [6]. Despite LFD’s good therapeutic results, its long-term oral administration is related to several gastrointestinal side effects, such as diarrhea, nausea, and dyspepsia, as well as liver toxicity [7,8,9]. Hence, in order to reduce these adverse events, several studies assessed the preparation of topically administrated TFL (the active metabolite) [10,11]. For example, Xi et al. reported the development of a transdermal patch for the intra-articular delivery of TFL through the skin [11], while Siafaka et al. prepared novel electro-spun nanofibrous matrices for TFL’s skin delivery [10]. However, despite these encouraging results, it is important to note that, in almost all of the previous studies reported, TFL (i.e., the active metabolite) was used instead of LFD. The main reason for this was probably the fact that the bioconversion of LFD into TFL (the active metabolite) across the skin was unknown. This matter, however, was recently resolved by Bae and Park, who showed that topically applied LFD can be delivered effectively and deposited as TFL in an arthritic joint [12]. Specifically, in this recent study, the use of topical LFD showed 3.4-fold to 54.6-fold higher concentrations of TFL in the knee joint compared to oral administration. Hence, based on these findings, the use of LFD instead of TFL may be an attractive solution for the preparation of an effective topical RA treatment (leading to fewer side effects and better patient compliance). However, and despite the above promising result, the preparation of a skin-delivery LFD patch is not problem-free, since there are several problems associated with LFD’s skin permeability and aqueous solubility [13]. In this vein, for the preparation of an effective LFD topical delivery system, new and novel formulation approaches should be adopted. 

Chitosan (CS) is a hemi-synthetic cationic linear polysaccharide [14]. It is synthesized by the deacetylation of chitin, which is a naturally occurring polysaccharide. It is an easily accessible polymer, but most importantly it is non-toxic, highly biocompatible, and biodegradable, with low immunogenicity, and, as such, it is an excellent candidate for pharmaceutical and biomedical applications such as transdermal patches [14,15,16]. Furthermore, CS nanoparticles (NPs) can pass through tight junctions of skin and deliver drugs to the capillary blood vessels and finally to blood circulation. Al-Kasas et al. prepared CS NPs by ionic gelation in order to be test their skin penetration, where they found that NPs prepared with gum arabic were able to penetrate porcine ear skin, showing their ability to deliver the drug transdermally [17].

Additionally to CS, for the preparation of skin patches, several polymeric biodegradable materials have been utilized until now (either alone or in combination with other suitable carriers), including poly(amides), poly(amino acids), poly(alkyl-a-cyano acrylates), poly(esters), poly(orthoesters), poly(urethanes), and poly (acrylamides) [18,19,20]. Amongst them, aliphatic polyesters (prepared from either lactic or glycolic acid, or a combination of them) are probably the most widely used polymeric materials for this reason [21,22,23]. Specifically, poly(l-lactic acid) (PLLA), a biodegradable thermoplastic polyester derived from renewable plant sources, such as starch and sugar, is an extensively used in biomedical applications and especially in the preparation of dermal or transdermal formulations due to its unique characteristics (such as slow degradation, good adhesion, elasticity, etc.) [24]. Similarly, poly(lactic-co-glycolic acid) (PLGA) is also widely used for the preparation of skin delivery pharmaceutical formulations, since it offers more highly tunable characteristics (such as degradation rates) compared to PLLA [25]. 

In recent years, the scientific community used the combination of NPs entrapped in hydrogel thin-films scaffolds in order to release the drug in a specific area with simultaneous wound healing effects. In brief, Guo et al. prepared silk fibroin hydrogel scaffolds containing CS NPs, resulting in chondrogenesis of articular cartilage defects [26]. Similarly, Shafique et al. prepared a hydrogel membrane using hyaluronic acid and CS NPs for the delivery of cefepime, a drug used as a parenteral antibiotic [27]. Wound healing in a rat model was observed due to a hydrogel membrane group increase due to hyaluronic acid, resulting in faster healing, while cefepime’s sustained release observed form CS NPs prevented the wound from infection. Additionally, other recently published novel studies used CS NPs and their incorporation in polyvinylpyrrolidone nanofibers as drug delivery system without the need of a wound healing agents. In brief, Tuğcu-Demiröz et al. encapsulated benzydamide, used in vaginal infections, in CS NPs and further dispersed them in PVP nanofibers by electrospinning [28]. TEM images showed that CS NPs were successfully dispersed in the nanofibers, retaining also their size without showing any agglomeration. Furthermore, CS Ps increased mucoadhesion properties of the patch prepared, as compared to the nanofibers prepared with the drug alone. Finally, nanofibers with CS nanoparticles showed an enhanced release profile while permeability of the drug was also higher.

Therefore, based on the above set framework, the present work focuses, for the first time, on the preparation of a suitable controlled-released LFD-loaded skin patch, utilizing the combination of CS NPs incorporated into suitable PLLA or PLGA matrices. Specifically, after the preparation and characterization of LFD-loaded CS NPs, suitable PLLA- or PLGA- based thin-film skin patches will be prepared via spin coating, and their physicochemical and in-vitro drug release properties will be thoroughly evaluated. 

## 2. Materials and Methods

### 2.1. Materials

A high purity LFD drug substance was kindly donated by Pharmathen S.A. (Athens, Greece). Low molecular CS (5–19 kDa), low-hydrolyzed (87–89%) polyvinyl alcohol (PVA), and triphenyl phosphate (TPP) were purchased from Aldrich Chemical Co. (Stainheim, Germany). PLLA with a molecular weight of 63,090 g/mol and PLGA (at a 75/25 and 50/50 ratio of lactic to glycolic acid) were purchased from Corbion (Gorinchem, The Netherlands). All other reagents were of analytical or pharmaceutical grade and used as received.

### 2.2. Preparation of NPs

The preparation of CS NPs in the present study was based on a modification of the previously published ionotropic gelation method [29,30,31]. Briefly, blank NPs were prepared with the addition of a TPP aqueous solution (2 mg/mL) to a CS acetic acid solution (2% *v*/*v* of CS to acetic acid). The concentration of acetic acid was in all cases 1.75 times higher than that of CS. The CS/TPP ratio was set at 4:1 *w*/*w*. During ionotropic gelation, the formation of NPs is the result of the interaction between the negative groups of TPP, and the positively charged amino groups of CS. For the preparation of drug-loaded NPs. a modified ionic gelation method was used [32]. In brief, after dissolving the CS amount in 20 mL acetic acid solution (2% *v*/*v* in concentration), an aqueous solution of 1.0% *w*/*w* of PVA was inserted while, immediately after, LFD that was dissolved in dichloromethane (10 mg/mL in concentration) was added, and probe sonication took place for 1 min. The resulting emulsion was placed under magnetic stirring, and the TPP aqueous solution (2 mg/mL) was added dropwise. The final suspension was left under mild magnetic stirring for 24 h. The formed NPs were collected by centrifugation at 12,500 rpm for 20 min at an ambient temperature. In all cases, the drug-loaded or neat NPs collected were resuspended in pure water, frozen, and lyophilized via a Scanvac freeze drier system (Scanvac Coolsafe, Labogen Scandinavia, Lillerød, Denmark) for 12 h at −56 °C. Τhe resulting dried NPs were hermetically sealed and placed in a desiccator until further use. All trials were conducted in triplicate.

### 2.3. Characterization of NPs

#### 2.3.1. Fourier-Transformed Infrared Spectroscopy (FTIR)

The obtained CS NPs as well as the raw materials were evaluated by FTIR spectroscopy (model FTIR-2000, Perkin Elmer, Waltham, MA, USA). Potassium bromide was used for the formation of suitable disks before the analysis. All spectra were collected from 400 to 4000 cm^−1^ with 4 cm^−1^ resolution (64 co-added scans), and the baseline was corrected and converted into absorbance mode.

#### 2.3.2. Wide-Angle X-ray Scattering (XRD)

For the powder XRD (pXRD) analysis of the obtained samples and the raw materials, a Rigaku XRD-diffractometer was used (Miniiflex II, Chalgrove, Oxford, UK). CuKα radiation was used for crystalline phase identification with λ = 0.15405 nm. A range of 5 to 55° was selected with a speed of 1 °/min.

#### 2.3.3. Differential Scanning Calorimetry (DSC)

A Perkin–Elmer Pyris 1 differential scanning calorimeter (Perkin-Elmer, Waltham, MA, USA) was used for the DSC analysis of the samples and the raw materials. A small accurately weighted quantity of each sample (~10 mg) was placed in sealed aluminum pans and initially heated to approximately 105 °C (with 20 °C /min) held for 10 min in order to remove the absorbed water, then quenched cooled to approximately 0 °C (with ~200 °C/min) and heated again up to 200 °C. In order to erase the thermal history, the samples were held at 200 °C for 5 min before quenching again to 20 °C and reheating to 200 °C (with 20 °C/min). All trials were performed in an inert atmosphere (N_2_, flow rate 50 mL/min), while the instrument was calibrated with Indium and Zinc standards.

#### 2.3.4. Field Emission Scanning Electron Microscopy (FESEM)

FESEM images were acquired with an electron microscope JEOL JSM-7610F Plus supported by an Oxford AZTEC ENERGY ADVANCED X-act energy dispersive X-ray spectroscopy (EDS) system (JEOL, Tokyo, Japan). Briefly, for the preparation of the samples, a small quantity of each of NPs’ suspension was dropped on a suitable holder and, after evaporating, was covered with carbon. During the measurements, the accelerating voltage was set at 20 kV, and a probe current of 45 nA was selected, while the counting time was set at 60 s.

#### 2.3.5. High-Pressure Liquid Chromatography (HPLC)

Quantitative analysis and drug loading were performed using a Shimadzu HPLC (Shimadzu, Kyoto, Japan) prominence system previously described [10]. The analytical column was a CNW Technologies Athena C18, 120 A (5 μm, 250 mm × 4.6 mm), and the analysis was performed at 25 °C. Acetonitrile/water (pH = 3.0 using phosphoric acid) 80/20 *v*/*v* was used as a mobile phase with a flow rate of 1.0 mL/min and 20 μL injection volume. The quantification of LFD was performed using a UV detector at 295 nm. The calibration curve was created by diluting a stock methanol solution of 500 ppm LFD to concentrations of 0.01, 0.025, 0.05, 0.1, 0.25, 0.5, 1.0, 2.5, 5.0, 7.5, 10.0, and 25.0 ppm.

For the determination of NP’s yield, drug loading, and encapsulation efficiency (EE), 10 mg of the prepared NPs were dissolved in 10 mL of aqueous acetic acid solution (1% *v*/*v*)/methanol (50:50 *v*/*v*). The resulting solution was stirred for 24 h and filtered (nylon filters, 0.45 nm pore size). NP’s yield, drug loading, and encapsulation efficiency (EE) were evaluated by applying the following Equations:

NP’s Yield (%) = (Weight of NPs)/(Initial weight of polymer and drug) × 100 (1)

Drug Loading (%) = (Weight of drug in NPs)/(Weight of NPs) × 100(2)

EE (%) = (Weight of drug in NPs)/(Initial weight of drug) × 100(3)

#### 2.3.6. Molecular Dynamics (MD) Simulations

MD simulations were used in order to evaluate the molecular interactions occurring between the drug (LFD) and the polymeric carrier (CS) during the NPs’ preparation in depth. 

*Initial structures*: The initial molecular structure of CS was prepared using VEGAZZ v.3.1.1.42 [33] based on similar, previous studies [34,35,36]. A high degree of deacetylation (83%) was selected during the construction of the polymer chain which included 50 monomers. LFD molecular structure was obtained from pubchem (CCDC No: 259170) retrieved from the work of Vega et al. [37]. After setting the initial molecular structures, both compounds were treated as non-periodic structures and optimized via energy minimization using the *pcff_d* force field [38] with XenoView v. 3.8.1.0 molecular modeling software (http://www.vemmer.org/xenoview/xenoview.html, accessed on 30 November 2020). Atomic charges were assigned by the *pcff_d* model. The steepest descent algorithm was used during geometry optimization (tolerance limit and maximum displacement per iteration were set at 1.00 × 10^−4^ kcal/mol and 10 Å, respectively). Ten structures were optimized in total, and the one with the lowest energy was selected for further processing. 

*Amorphous cell*: The Xenoview amorphous build tool was utilized to prepared the amorphous assemblies of CS-LFD at 298.15 K (25 °C) by varying the rotatable torsions using the free rotation model method. The amorphous cell contained one CS polymeric chain with 100 monomers and four LFD molecules. The cell structure was further equilibrated following a multistep equilibration protocol proposed by Li et al. [39]. 

*MD simulations*: The optimum molecular assembly was further equilibrated with periodic boundary conditions for approximately 1.0 ns (1 fs time step) at 608.15 K (at 1 atm). A cut-off radius of 10 Å and a spline distance of 1 Å were selected. The variable volume and shape option was used under Berendsen’s thermostat. Then, the molecular structures were subjected to a cooling up to 200 K (with 10 K step) under NPT conditions. The final configuration of the molecular dynamics run was used as the starting structure for the next stage of the subsequent run in each temperature. A final MD simulation run (1.0 ns at 1.0 atm) was performed at 298.15 K (25 °C) using a cut-off radius of 10 Å, along with a spline distance of 1 Å, a Berendsen thermostat, and a barostat, as well as a 1.0 fs time step. The parameters proposed by van Gunsteren and Mark for model creation, force field selection, sampling scheme setup, and software selection/use were used to ensure the simulation’s validity [40]. 

The radial distribution function, *g*(*r*), was calculated in order to gain in-depth information on the drug and polymer molecular interactions. *g*(*r*) describes the correlation between atoms in a given system and is calculated by the following equation:

(4) $$g\left(r\right)=\frac{V\sum_{i\neq j}\delta \left(r-\left|r_{Ai}-r_{Bi}\right|\right)}{\left(N_{A}N_{B}-N_{AB}\right)4\pi r^{2}dr}$$

where *A* and *B* are specific atoms; *V* is the system volume; *N_A_* and *N_B_* are particle number of atoms *A* and *B*, respectively; *N_AB_* are the number of particles belonging to atom *A* and atom *B* simultaneously; and *r_Ai_* and *r_Bj_* represent the position of particle *i* of atom *A* particle *j* of atom *B*, respectively.

### 2.4. Preparation of Thin-Film Patches via Spin-Coating

Patches in the form of PLLA and PLGA thin-films were prepared using spin-coating (Laurell WS-650-23, Laurell Technologies Corporation, North Wales, PA, USA) according to the following procedure. Initially, in order to identify the optimum spin-coating conditions, PLLA or PLGA was dissolved in 20 mL of dichloromethane (using magnetic stirring for 2 h at room temperature) until a clear solution was formed. The resulting solution was spin-coated on silicon substrates in order to produce thin-film patches. Several polymer concentrations (5%, 10%, and 20% *w*/*w*) and angular velocities (2000, 2500, 3000, 4000, and 6000 rpm) were tested in order to optimize the spin coating process. All trials were conducted at a 500 rpm/s acceleration for 1 min. Then, the optimum-identified conditions were used for the preparation of all PLLA and PLGA thin-films. In the case of LFD-loaded films, the pure drug was weighted with the proper amount of PLA, or the PLGA, and then dissolved in dichloromethane, and the same spin-coating procedure was followed as described above. Table 1 summarizes the concentrations of the LFD-loaded thin-film patches prepared in the present study.

The selection of PLLA and PLGA concentrations up to 80 wt.% is based according to previous reports utilizing similar polyester for the preparation of biodegradable thin-film patches [41,42,43]. The use of higher polymer concentrations leads to higher drug content, which in turn means that the properties of the final skin patch will not be controlled by the polymeric matrix, leading to a significant loss in the biodegradation and sustained drug release properties of the optimum thin-film patch. 

In the case of LFD-loaded CS NPs, the thin-films were prepared by dispersing the NPs in the PLLA or PLGA dichloromethane solutions (0.1% or 0.5% *w*/*w* of NPs to PLLA or PLGA were used) followed by a similar spin-coating process as previously described. In all cases, the resulting thin-films were cut into 2 cm × 2 cm square pieces and stored in desiccants before further use. Drug loading and EE in the prepared thin-films were calculated using the equations described previously (Section 2.3.5). It is important to note that the weight ratio of 5, 10, and 20% *w*/*w* for LFD was selected, taking into account the available commercial drug products’ doses (i.e., 10, 20, and 100 mg for daily oral use). The selection of these proper concentrations made it possible to simulate the drug content depending on the area of prepared patches. Additionally, in the case of drug-loaded CS NPs, the selection of the drug content was made based on the final morphology of the patch.

### 2.5. Characterization of Patches

FTIR, XRD, DSC, drug loading, and EE analyses of the prepared thin-film patches were performed by following the same experimental procedure as described in Section 2.3. 

#### In Vitro Dissolution Studies

For the in-vitro release studies, a DISTEK Dissolution Apparatus I (North Brunswick, NJ, USA) equipped with an autosampler was used. The sample was held in appropriate transdermal patch holders which were purchased from the QLA Company (Telford, Pennsylvania, USA). Dissolution was performed at 37 ± 1 °C, and the rotation speed was set at 50 rpm. The dissolution medium was 300 mL of simulated body fluid (SBF) at pH = 7.4 with 0.1% *v*/*v* of Tween 20. It should be noted that SBF with pH = 7.4 was selected because the final target for the drug delivery is the body fluids. Although it is known that dermal pH is varying from 5.2 to 5.4, without showing any difference to its value in psoriatic skin, the formulations prepared are intended to deliver the drug through the skin to capillary blood vessels. Two milliliters of aqueous solution were withdrawn from the release media at predefined time intervals (5, 15, 30 min and 1, 3, 6, 12, 24, 36, 48, 60, 72, 96, and 120 h) and quantified via the HPLC method described previously in Section 2.3.5.

### 2.6. Statistical Analysis

Statistical significance in the differences between the group means was evaluated by using Student’s *t*-test. The SPSS statistic software (SPSS version 15.0, SPSS Inc., Chicago, IL, USA) was used. A difference with a *p*-value (*p* *) < 0.05 was considered statistically significant. Additionally, for the comparison of the dissolution profile, the similarity factor (*f*_2_) was used. The *f*_2_ is a logarithmic transformation of the sum-squared error of differences between the test and reference over all time points [44]:

(5) $$f_{2}=\left[{\left(1+\frac{1}{n}\overset{n}{\underset{t=1}{\sum }}{\left(R_{i}-T_{i}\right)}^{2}\right)}^{-0.5}\times 100\right]$$

where, *R_t_* and *T_t_* represent the cumulative percent dissolved for the two test samples at each time point to be compared, and *n* is the number of dissolution time points. The *f*_2_ value can take values between 0 and 100, with zero revealing complete dissimilarity and 100 identicality. Two dissolution profiles are considered to be similar when the *f*_2_ value is larger than 50 (meaning that the mean deviation over all time points was less than 10%).

## 3. Results and Discussion

### 3.1. LFD-CS NPs

Initially, before the preparation of the thin-film patches, LFD was encapsuled in CS-based NPs in order to overcome the several problems deriving from the unfavorable physicochemical properties of the drug.

NPs were prepared according to a modified ionic gelation technique in which interactions are taking place between the negative groups of TPP and the positive charged tertiary amino groups of CS while PVA is further used in order to enhance the solubility of the hydrophobic drug. The formation of CS/TPP NPs was already studied from our group by an incorporation of timolol for ocular delivery, along with annatto or saffron for UV protective cosmetic emulations, as well as for the preparation of budesonide NPs [45,46,47,48]. Additionally, in a previous study of ours, the effect of the CS/TPP ratio was evaluated thoroughly, showing that the utilization of a 4:1 *w*/*w* ratio results in the optimum (smallest) NPs’ size [49]. Therefore, since in the scope of the present study, the preparation of LFD skin patches and NPs with the smallest achievable particle size is most desirable (in order to increase drug’s wetting properties), the same 4:1 *w*/*w* CS/TPP ratio was also used. LFD-loaded CS NP prepared in this way shows adequate drug loading (18.06 ± 0.92%), good yield (23.54 ± 2.85%), and EE (42.08 ± 1.64%).

**Figure 1:**
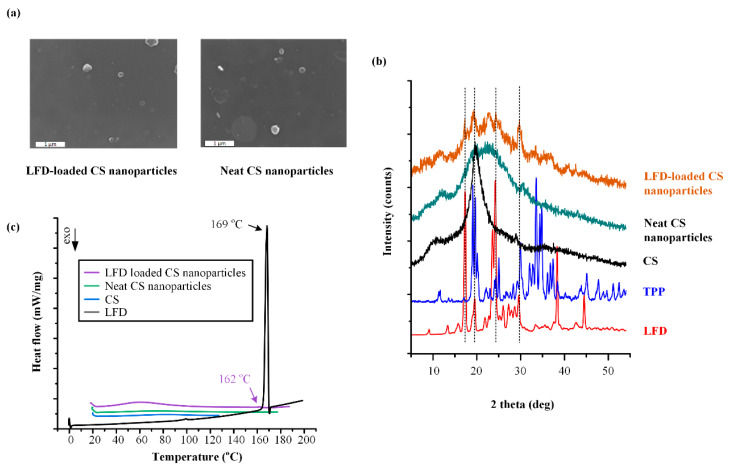
FESEM images (**a**), pXRD diffractograms (**b**), and DSC thermograms (**c**) for the neat chitosan (CS) nanoparticles (NPs) and the leflunomide (LFD)-loaded CS NPs that will be embedded into the poly(l-lactic acid) (PLLA) and poly(lactic co glycolic acid) (PLGA) thin-film patches.

Figure 1a shows the FESEM images of the LFD-loaded NPs, as well as the images of the neat CS NPs (i.e., without the use of LFD). As it can be seen, neat CS NPs had a smooth surface and an almost spherical shape, while their average particle size (i.e., d_50_) was 380 nm. On the contrary, LFD-loaded CS NPs showed a less spherical shape with a smooth surface and an average particle size of approximately 600 nm. In both cases, no particle aggregation was observed.

In a further step, the changes in the physical state (i.e., crystalline or amorphous) of the LFD during the preparation of the drug-loaded NPs were evaluated with the aid of pXRD (Figure 1b). Pure LFD is a highly crystalline compound showing pXRD characteristic peaks at 2θ of 17.0, 19.6, 23.6, 24.2, 38.4, and 44.6 deg. Similarly, TPP is also highly crystalline with characteristic diffraction peaks at 2θ of 19.1, 19.7, 25.1, 29.9, 33.6, 34.9, 36.9, and 37.5 deg. In contrast, CS is a semi-crystalline polymeric material with two characteristic diffraction peaks at 2θ of 11.0 and 21.0 deg [50]. In the case of NPs, Figure 1b shows that there is a clear reduction in CS’s crystalline phase in both the neat and the LFD-loaded NPs. This may be attributed to the rearrange of the intermolecular and intramolecular network of the CS polymer, resulting from the crosslinking reaction with the TPP ions (a more comprehensive evaluation on the formation of molecular interactions between CS and TPP will follow during the discussion of the FTIR results). Furthermore, the intensity of the characteristic peaks of the drug is significantly reduced, indicating that the process followed results in the partial amorphization of LFD within the formed CS NPs. Analogous observations were also found in our previous studies after NPs’ formation with chitosan and TPP for nanoencapsulation of desferrioxamine mesylate [51], where the reduction in CS peaks showed the amorphous state of the entrapped substance. 

In order to verify the XRD findings regarding the physical state of the drug, as well as to evaluate possible interactions between the drug and the polymer, DSC studies were also conducted. Figure 1c shows thermographs of the pure drug, the pure CS, as well as the neat and the drug-encapsulated NPs. With regard to neat CS, the obtained thermograms showed a broad endothermic peak at around 90 °C, which is probably attributed to the removal of water adsorbed or bound to chitosan [47], while the DCS thermogram for the pure LFD showed a sharp endothermic peak at 169 °C, corresponding to the melting of LFD crystals. Neat CS NPs showed a similar thermal pattern as the neat CS, while the prepared LFD-loaded NPs showed the presence of a small endothermic peak at 162 °C, corresponding to the melting point of the drug. Hence, based on the above results, the obtained DSC thermograms verify that LFD dispersed within the CS NPs was, at least partially, crystalline. 

**Figure 2:**
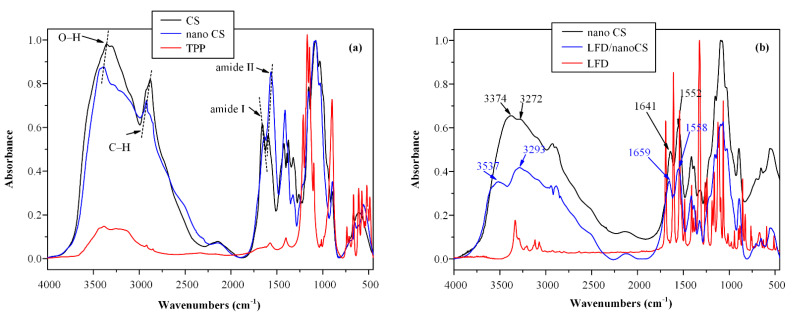
FTIR spectra of: (**a**) neat CS, neat triphenyl phosphate (TPP) and neat CS NPs (nano CS), and (**b**) neat LFD, neat CS and LFD-loaded CS NPs (LFD/nano CS).

In a further step, since, during the preparation of drug-loaded CS NPs, a physicochemical interaction may take place among the several components, FTIR analysis was used in order to examine the possible interactions between CS and TPP as well as CS and LFD. Figure 2 shows the FTIR spectra of the neat CS, TPP, and LFD compounds as well as the neat CS and the LFD-loaded NPs. 

With regard to CS, FTIR analysis showed a broad characteristic peak in the region of 2500–3800 cm^−1^ attributed to O–H and N–H stretching vibration peaks, two peaks at 1641 and 1558 cm^−1^ attributed to the amide I and II, respectively, a peak at 1324 cm^−1^ corresponding to the axial deformation of the C–N bond, and two peaks at 1074 and 1026 cm^−1^ attributed to the C–O bond of cyclic alcohols and to the primary hydroxyl groups of the polysaccharide [50]. LFD characteristic FTIR peaks were observed at 3333 cm^−1^ attributed to a NH peak of amide, a sharp doublet peak appearing at 2924 cm^−1^ assigned to a CH stretching vibration, a sharp peak at 1690 cm^−1^ due to the HC=N–O of the isoxazole ring, a sharp peak at 1604 cm^−1^, a peak at 1540 cm^−1^ assigned to C=O of the amide, and a peak at 1504 cm^−1^ attributed to C=C stretching vibrations. Additionally, TPP showed characteristic FTIR peaks at 3059 cm^−1^ corresponding to the C-H stretching vibration in the region of 1454–1589 cm^−1^ corresponding to the C=C stretching vibration, at 1294 cm^−1^ corresponding to P=O stretching in the region of 850–1010 cm^−1^ corresponding to the P–O stretching vibrations, and in the region of 690–786 cm^−1^ corresponding to the C–H deformation. Looking at the FTIR spectrum of the neat CS NPs, several shifts in the characteristic absorbance peaks of the CS amides (both I and II) and the peaks corresponding to the phosphate group of TPP (allocated at 897 cm^−1^) are observed, indicating the formation of molecular interactions between the amino groups of the CS polymer with the phosphate groups of TPP [50,52]. In the case of LFD-loaded CS NPs, results show significant shifts in the region of 3540 to 3290 cm^−1^ and 1660 to 1550 cm^−1,^ indicating the formation of molecular interactions between the amino or the hydroxyl groups of CS and LFD, probably via hydrogen bonding (HB). 

Based on the above results and the FTIR and DSC evaluation of the LFD-loaded CS NPs, it was found that interactions are taking place between the drug and the CS polymer. However, it was impossible to evaluate the extent and the nature of these interactions. Therefore, in order to identify the type of these interactions more precisely and to uncover the underlying mechanisms for their formation, MD simulations between the two components (i.e., the drug and the polymer) were conducted as described in Section 2.3.6. 

**Figure 3:**
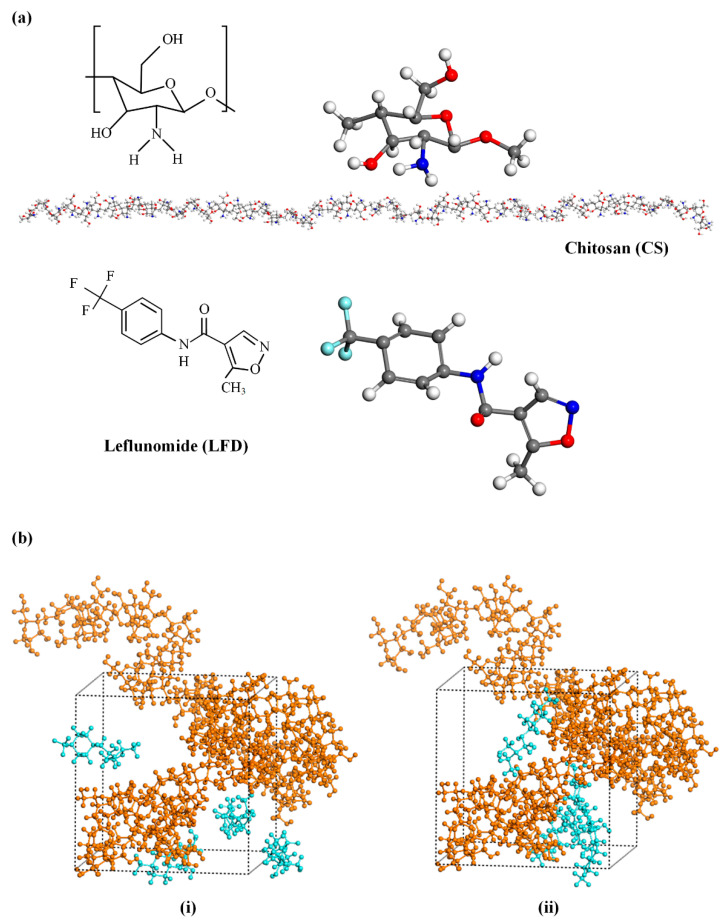
(**a**) Initial MD chemical structures of the CS polymer with 50 monomers and the LFD drug molecule (carbon atoms are depicted with grey, hydrogen with white, nitrogen with blue, oxygen with red, and fluoride with blue). (**b**) MD simulation boxes for LFD (blue color) and CS (orange color) mixture, before (**i**) and after (**ii**) the adoption of the multistep equilibration protocol.

The initial molecular structures and the conformations of the amorphous cells before and after the multistep MD equilibration protocol of the CS-LFD assembly are shown in Figure 3a,b, respectively. Results indicate that the implementation of the multistep equilibration protocol results in a much more homogeneous amorphous structure compared to the initially obtained assembly. With regard to drug-polymer interactions, looking at the molecular structures of the CS monomer and LFD molecule given in Figure 3a, it is obvious that both components possess several molecular groups that can act as HB donors or acceptors (for example –NH, –C=O, –OH, –C–O–C–, etc.). Therefore, during the preparation of drug-loaded CS NPs, HB interactions between the two components may be formed. 

**Figure 4:**
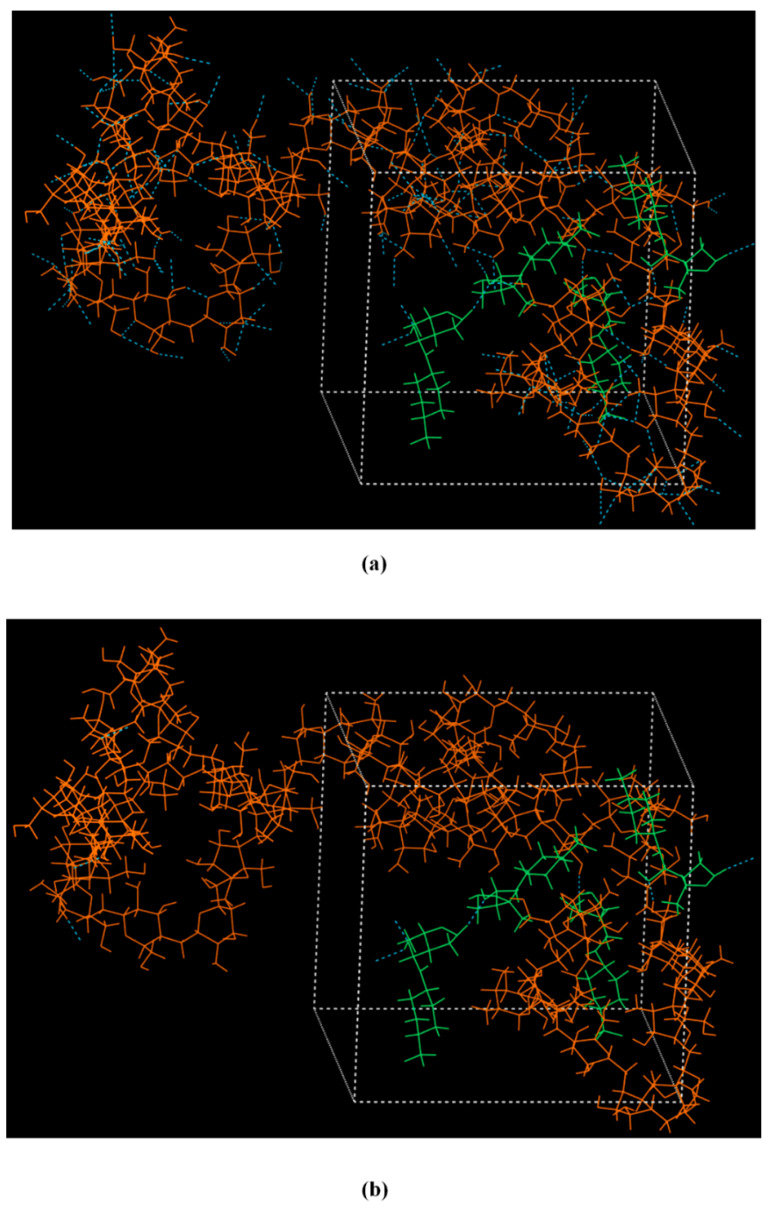
(**a**) Amorphous CS-LFD structures showing: (**a**) the formation of HBs among the neat CS molecules, and (**b**) the formation of HBs between the CS and LFD molecules (CS is depicted with orange and LFD with green).

Figure 4a,b shows the HB patterns obtained for the amorphous CS-LFD structure after the completion of the 1.0 ns MD simulations. The maximum hydrogen-acceptor distance was set at 2.5, and the minimum donor-hydrogen-acceptor angle was set at 90^o^ for the measurement of HBs. Results presented in Figure 4a show that the majority of the HBs are among the neat CS molecules; however, there are several HBs formed between the neat LFD molecules as well as between the drug and polymer molecules (Figure 4b). Therefore, based on the MD simulations, during the formation of CS-LFD NPs, both hetero- (i.e., CS-LFD) and homo- (i.e., CS-CS and LFD-LFD) HBs are being formed.

**Figure 5:**
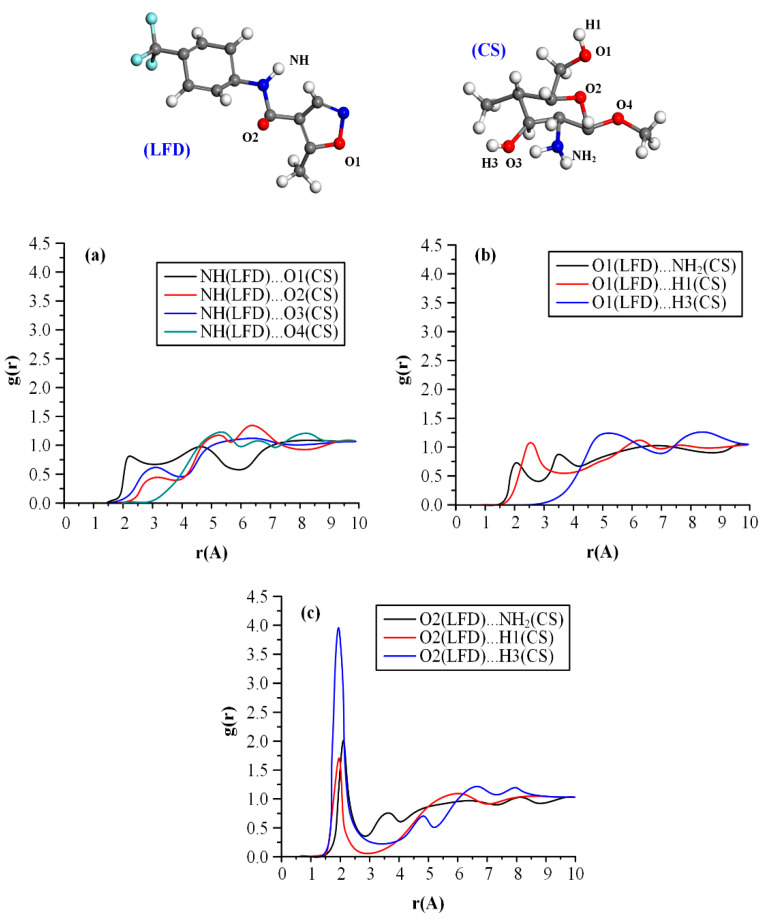
Radial distribution function, g(r), between LFD’s –NH proton donor H1 with CS’s HB acceptors O1–O4 (**a**) as well as LFD’s proton acceptors O1 (**b**) and O2 (**c**) with CS’s HB proton donors from the –NH2 and the –OH (H1 & H3) groups.

The type of hetero-intermolecular interactions involved during the CS-LFD NPs preparation processes were evaluated with the aid of the radial distribution (or pair correlation) function, g(r), as shown in Figure 5.

In general, g(r) gives a measure of the probability that an atom with its center located in a spherical shell is placed at a distance r regarding another reference atom. Peaks with donor-acceptor distances below 2.5 Å indicate strong, mostly covalent interactions; distances between 2.5 and 3.2 Å indicate moderate, mostly electrostatic, interactions, while distances between 3.2 and 4.0 Å indicate weak electrostatic interactions. Figure 5 shows the MD estimated g(r) profiles for the CS-LFD molecular assembly. Results showed three strong g(r) peaks corresponding to O2(LFD)…NH2(CS), O2(LFD)…H1(CS), and O2(LFD)…H3(CS) interactions at 2.19 Å, 2.05 Å, and 2.09 Å, respectively, indicating significant HB interactions between the drug and the polymer. Specifically, based on the obtained results, the strongest g(r) peaks were obtained for the O3 oxygen of the drug, indicating that the most significant intermolecular interactions between LFD and the polymer are occurring from the formation of significant HBs between LFD’s carbonyl oxygen (O2) and CS’s HB donors coming from polymer’s –NH2 and –OH groups.

In sum, based on the above analysis, LFD was successfully encapsulated within the CS matrices, forming in this way NPs with acceptable drug loading and EE, while the obtained nanoparticles’ size was reduced enough in order to enhance LFD’s wetting properties when embedded into the polyester thin-film patches (this hypothesis will be tested during the in vitro dissolution evaluation of the thin-film patches) 

### 3.2. PLLA and PLGA Thin-Film Patches

#### 3.2.1. Optimization of Spin-Coating Process

Initially, before proceeding with the preparation of LFD-loaded films, the spin-coating process was optimized in terms of polymer concentration (5%, 10%, and 20% *w*/*w*) and angular velocity (2000, 2500, 3000, 4000, and 6000 rpm). In the case of PLLA polymeric films, results on process optimization showed that increasing polymer concentration above 10% *w*/*w* led to the formation of polymer agglomerates within the thin-film structures as well as a small spread radius and trapped air, while the increase in angular velocity led to the formation of fragmented polymeric thin-films. On the contrary, in the case of PLGA, increasing polymer concertation did not deteriorate the quality of the produced film, while as in the case of PLLA, increasing angular velocity led to the preparation of fragmented PLGA films. Therefore, based on the optimization results, the optimum conditions for the preparation of PLLA and PLGA thin-films, 10% and 20% *w*/*w* of polymer concentration were selected, respectively, while in both cases a low angular velocity (i.e., 2500 rpm) was selected as optimum.

#### 3.2.2. LFD-loaded Thin-Film Patches

After identifying the optimum polymer concertation and spin-coating parameters, polymeric thin-film patches were prepared containing the neat LFD (i.e., the drug not encapsulated in the CS-NPs).

PLLA-based thin-film patches

In case of PLLA, the proposed working conditions were adequate enough in order to prepare LFD-loaded thin-film patches with good drug loadings and EE (Table 2).

**Figure 6:**
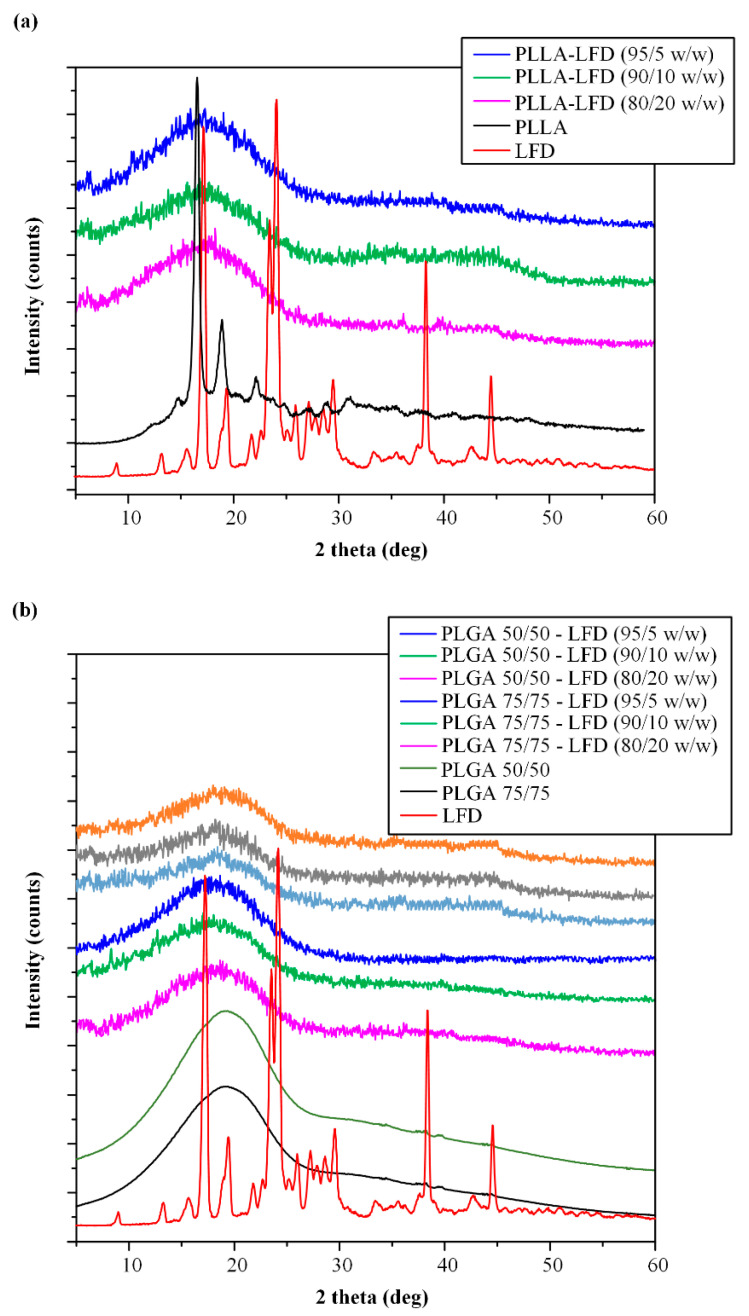
pXRD diffractograms of neat LDF-loaded PLLA (**a**) and PLGA (**b**) thin-film patches.

Changes in the physical state (i.e., crystalline or amorphous) of the drug during the preparation of the thin-film was evaluated with the aid of pXRD (Figure 6a). Pure LFD is a highly crystalline drug showing several characteristic pXRD diffraction peaks, as stated previously (Section 3.1). Similarly, PLLA is a semi-crystalline polyester showing characteristic diffraction peaks at 2θ of 16.6 and 18.96 deg. Results from the obtained diffractograms showed that, irrespectively of LFD’s content, the prepared LFD-loaded PLLA thin-films present a characteristic amorphous halo. This indicates not only that LFD was amorphously dispersed within the polyester film structure, but the polyester was also amorphous after the spin-coating process. This change in the physical state of both components indicates the possible formation of a molecular interaction between the drug and the polyester.

In a further strep, and in order to clarify whether molecular interactions are being formed between LFD and the polymer, the second heating DSC scans of the prepared PLLA patches and the neat components were collected. In order to do so, there are several theoretical and empirical equations describing the dependence of the glass transition temperature (T_g_) from the mass fractions and the T_g_s of the initial components. Among them, the Fox model described by the following equation is widely used in similar systems [53].

[1/Tg] = w_1_/Tg_1_ + w_2_/Tg_2_ (6)

where Tg is the glass transition temperature of the drug and polymer blend; w_1_ and w_2_ are the mass fractions of the PLLA and LFD that constitute the blend; and Tg_1_ & Tg_2_ are their respective glass transition temperatures. 

Similarly, Gordon and Taylor (GT) proposed a similar equation taking into account the possibility of significant interactions evolving between the studied components [53]:

Tg = (w_1_Tg_1_ + kw_2_Tg_2_)/(w_1_ + kw_2_) (7)

where k is a constant representing a semi-quantitative measure of the interaction strength between the reactive groups. If k takes values close to 1 or above, then strong interactions between the blend components are taking place [54,55].

**Figure 7:**
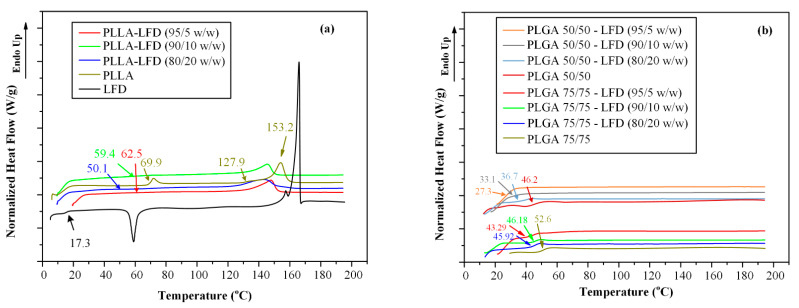
DSC thermograms of LDF-loaded PLLA (**a**) and LFD PLGA (**b**) thin-film patches after second heating scan.

Results from the second heating of DSC scans showing the true Tg values of samples are presented in Figure 7a. In the case of LFD, results showed a Tg value at 17.3 °C, followed by an exothermic crystallization peak and two overlapping endothermic melting peaks, respectively. Similarly, in the case of neat PLLA, a single Tg was recorded at 69.9 °C, followed by a small crystallization exotherm at 127.9 °C and a melting endotherm at 153.2 °C. Finally, in the case of LFD-loaded PLLA films, a single Tg was recorded irrespectively of LFD’s content in all cases, a strong indication that the two components (i.e., LFD and PLLA) are completely miscible. 

**Figure 8:**
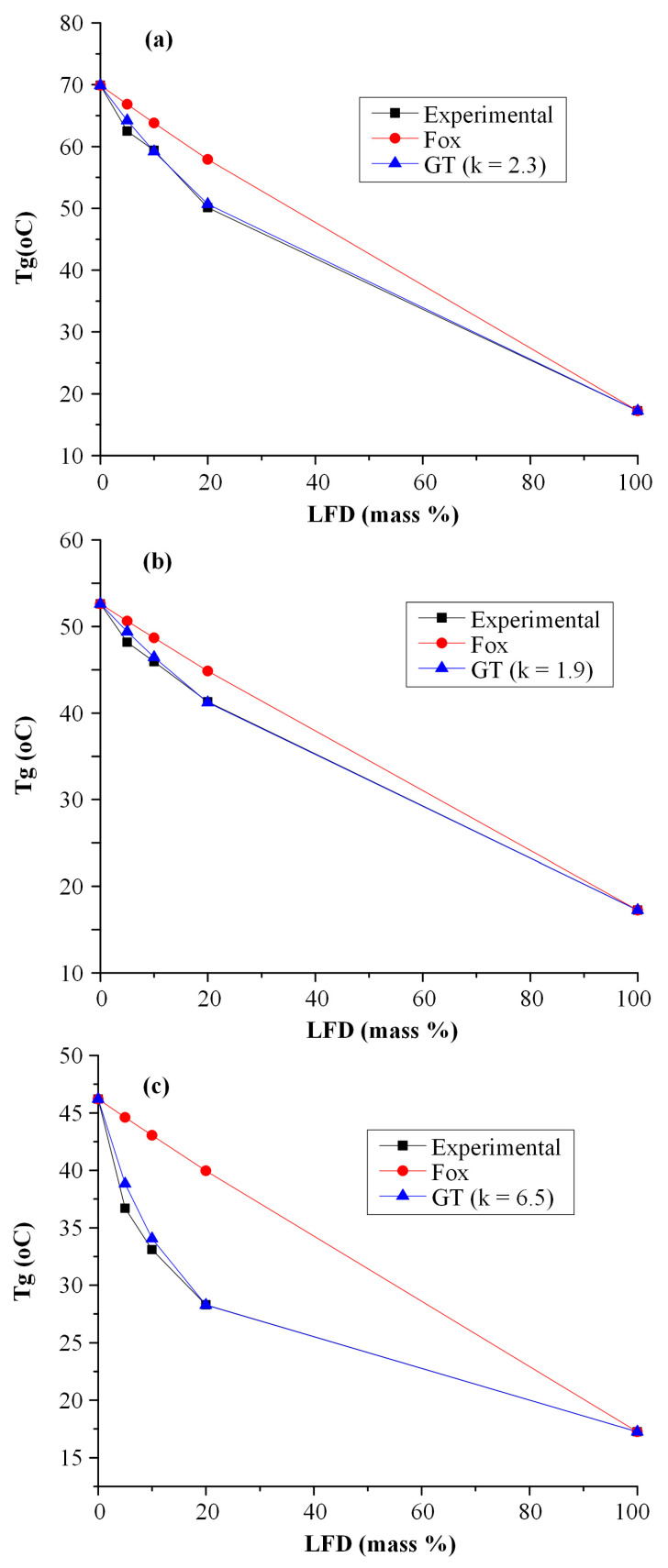
Prediction of Tg-composition dependence in LFD-PLLA (**a**), LFD-PLGA 75/25 (**b**), and LFD-PLGA 50/50 (**c**) thin-films by Fox and Gordon-Taylor (GT) equations.

Figure 8 shows the fitting results of Tg according to Fox and GT equations. Results showed that the theoretical predictions for Tg according to the Fox model are not in good correspondence with the recorded experimental values. This is because the Fox equation can be used only when no interactions are taking place. On the contrary, the theoretical predictions for Tg according to the GT equation fit quite well to the experimental data when the k parameter value is 2.3, indicating that significant intermolecular interactions are taking place between the two components.

**Figure 9:**
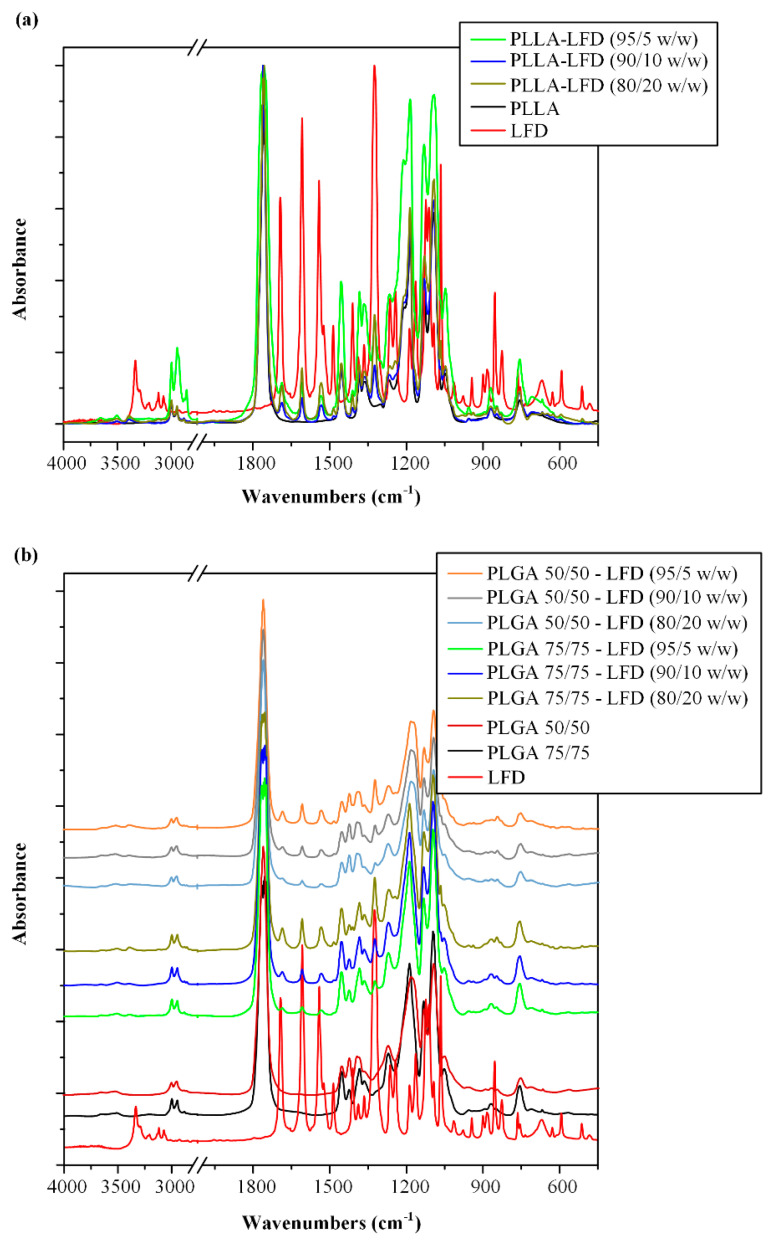
FTIR spectra of neat LDF-loaded PLLA (**a**) and PLGA (**b**) thin-film patches.

In a further step, in order to evaluate the type of these molecular interactions, the FTIR analysis was conducted. Figure 9a shows the FTIR spectra of the neat PLLA and LFD as well as the spectra of the LFD-loaded PLLA patches at several drug loadings.

With regard to neat PLLA, the FTIR analysis showed a characteristic double peak at 2998 and 2946 cm^−1^ due to –CH stretching vibrations and a characteristic peak at 1760 cm^−1^ due to –C=O stretching vibrations [56]. Similarly, the neat LFD showed several characteristic FITR peaks, all of them analyzed in Section 3.1. With regard to the LFD-loaded PLLA patches, results showed a shift in the –NH peak of the drug from 3333 to 3390 cm^−1^ as well as some minor shifts in LFD peaks attributed to the HC=N–O of the isoxazole ring and the –C=O amide stretching vibrations. These findings indicate the formation of a strong hydrogen bond formation between the –NH groups of the drug and the carbonyl or ether oxygens of the polymer, as well as some minor interactions between the carbonyl oxygen and the HC=N–O oxygens of the drug with the hydrogen protons located at the hydroxyl end groups of the PLLA polyester.

**Figure 10:**
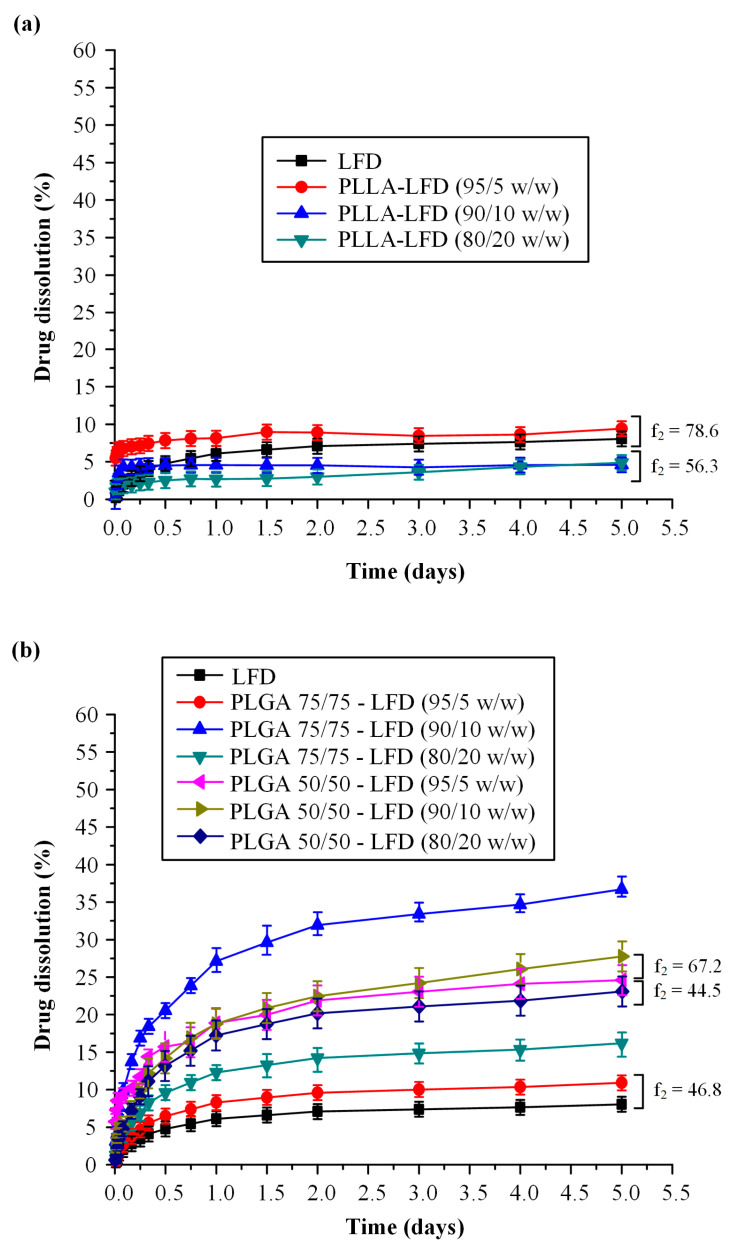
Dissolution release profiles in pH 7.4 of neat LDF-loaded PLLA (**a**) and PLGA (**b**) thin-film patches. Similarity factors (*f*2) results between the several dissolution profiles are also depicted.

Figure 10a shows the dissolution profiles of the LFD-loaded PLLA thin-films. The neat drug showed low drug release (not exceeding 10% even after five days of testing) which can be attributed to the high crystalline phase and the high hydrophobic nature of LFD. Similar behavior (i.e., low drug release) was also observed in all LFD-loaded PLLA thin-film patches, irrespectively of the drug content. Keeping in mind that the pXRD and DSC analysis of the samples showed that the drug was amorphously dispersed within the PLLA thin-films, the low dissolution profiles indicate that the dissolution process in all cases was probably controlled by the slow erosion rate (degradation process) of PLLA.

PLGA-based thin-film patches

As in the previous case, the selected spin-coating working conditions resulted in the production of the PLGA thin-films with good drug loading and EE values (Table 2). Similarly, changes in the physical state of the API were evaluated with the aid of pXRD (Figure 6b). In contrast to the neat PLLA (which is a semi-crystalline polyester), both PLGAs evaluated in the present study (i.e., 75/25 and 50/50) were completely amorphous. Similarly, all prepared LFD-loaded PLGA thin-films were amorphous, indicating that the API was amorphously dispersed within the polyester film structures. Therefore, as in the case of PLLA films, the change in the physical state of the API (i.e., from crystalline to amorphous) during the formation of the PLGA-based thin-films indicates the presence of a molecular interaction between the API and the polyester. 

In order to verify this hypothesis (i.e., the formation of molecular interactions between the two components), the second heating DSC scans were collected and analyzed (Figure 7b). In the case of neat polyesters, results showed a single Tg at 52.6 °C for PLGA 72/25 and 46.2 °C for PLGA 50/50, respectively, while in the case of LFD-loaded PLGA films, a single Tg was also recorded, indicating that the two components (i.e., LFD and PLGA 75/25 or PLGA 50/50) were completely miscible. As in the case of PLLA, the Tg analysis based on the Fox equation (Figure 8) were not in good agreement with the recorded experimental values, while the GT equation fitting showed quite good results when the k parameter value was set equal to 1.9 and 6.5 for PLGA 75/25 and 50/50, respectively. This indicates that significant intermolecular interactions between the two components are taking place during the preparation of the PLGA thin-films.

In order to identify the type of these DSC-suggested molecular interactions, the FTIR analysis was conducted. As in the case of PLLA drug-loaded films, results in Figure 9b showed similar shifts in the –NH, the HC=N–O, and the –C=O stretching peaks of the drug, indicating the formation of a strong hydrogen bond formation between the –NH groups of LFD and the carbonyl or ether oxygens of the polymer, as well as the formation of some minor interactions between the carbonyl oxygen and the HC=N–O oxygens of the API with the hydrogen protons located at the end groups of the PLGA polyesters.

Figure 10b shows the drug dissolution profiles from the obtained LFD-loaded PLGA thin films. Results show that in all cases, LFD’s release followed a two-step release process, i.e., an initial burst release up to 30 min (due to the drug being present in the surface of the films) and a controlled release up to five days. Additionally, the obtained results showed that in all PLGA films, much higher dissolution rates were achieved compared to the respective PLLA films. This enhancement can be attributed to the higher erosion (degradation) rate of PLGA compared to PLLA, since the drug, in both cases, was amorphously dispersed.

However, it is important to note that although the use of PLGA instead of PLLA resulted in a significant improvement in LFD’s dissolution rate and extent, in all studied cases, LFD’s release did not exceed 40%. This indicates that an improvement in the wetting characteristics of the drug is mandatory in order to improve its dissolution characteristics.

#### 3.2.3. LFD-Loaded CS NPs Emended on Thin-Film Patches

Based on the above results, and in order to improve LFD’s wetting characteristics, instead of the neat LFD, LFD-loaded CS NPs prepared in Section 3.1 were incorporated into the polyester thin-films.

**Figure 11:**
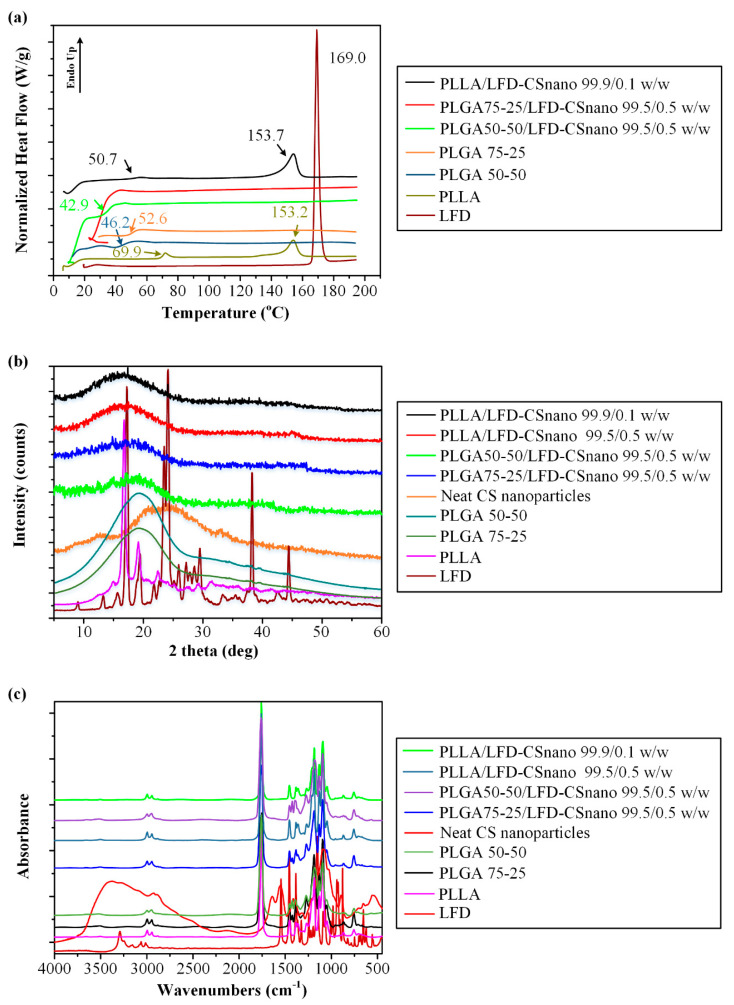
First heating DSC scan thermograms (**a**), pXRD diffractograms (**b**), and FTIR spectra (**c**) analysis of LFD-loaded CS-NPs emended in the PLLA and PLGA thin-films.

Figure 11a shows the DSC thermograms (first heating scan) for the LFD-loaded CS NPs embedded into the polyester thin-films.

Results suggest that in all cases, the drug was amorphously dispersed within the polyester matrices since no endothermic melting peaks attributed to LFD were recorded. The small endothermic peak observed at 153.7 °C in the case of PLLA-CS NPs can be attributed to the small portion of crystals remaining from PLLA. However, keeping in mind that LFD crystals present within the polyester-CS NP systems may not be detected from the DSC thermograms due to in situ amorphization (i.e., the drug crystals may melt within the DSC pan during the DSC heating process), the physical state of the drug within the prepared systems was also evaluated via pXRD.

Figure 11b shows the pXRD diffractograms for the LFD-loaded CS NPs embedded into the polyester thin-films. Results showed that in all cases, LFD was amorphously dispersed within the prepared matrix, thus verifying the DSC findings. Additionally, some small diffraction peaks were observed at 16.6 and 18.96 deg. In the case of the PLLA-CS NP thin-film, the presence of a small remaining portion of PLLA crystals within the system was revealed, a finding that is in agreement with the previously discussed DSC results. It is important to note that, in contrast to the results obtained in Figure 1 where drug crystallinity (a small portion) was observed in the pXRD diffractograms of the drug-loaded CS-NPs, results in Figure 11b show the complete amorphization of LFD within the polyester-CS NP system. This can be attributed to the small LFD content used in the later which resulted in a significant drop in the crystalline portion of the drug remaining in the system. In any case, this amorphization (or partial amorphization) of LFD within the prepared systems may be attributed to the presence of molecular interactions between the drug and the CS already discussed in detail in Section 3.1. In the case of drug-loaded polyester-CS NPs, these interactions, as well as the characteristic peaks of LFD and CS, were not seen in the obtained FTIR spectra (Figure 11c) which is a strong indication that the amount of LFD within the samples was below the detection limit of the method. 

**Figure 12:**
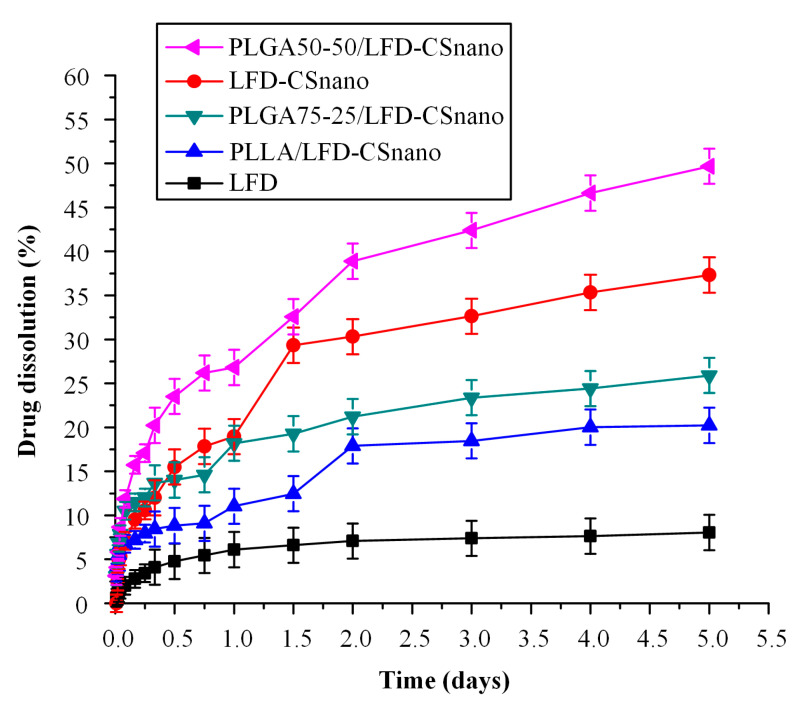
Drug release of LFD-loaded CS-NPs alone and emended in the PLLA and PLGA thin-films.

Figure 12 shows the drug dissolution release profiles from the LFD-loaded CS NPs and the same embedded into the PLLA and the PLGA thin films (at a ratio of 99.5/0.5 *w*/*w* polyester to LFD-CSnano).

Results showed a significant increase in both drug release rate and drug release extent in all cases (compared to the pure LFD) which can be partially attributed to the amorphous phase of the drug and partially to the increased wetting properties of the CS NPs. In all cases, a biphasic release profile was obtained. Specifically, a rather fast initial release was observed for up to approximately one day (which is useful to produce an immediate therapeutic effect) while a slow release was achieved for the remaining days (which is useful for the prolonged therapeutic effect of the LFD patches).

Compared to the dissolution results obtained from the neat LDF-loaded polyester thin-films (presented in Figure 10), the incorporation of LFD-loaded CS NPs brought about a critical increment of the cumulative amount of LFD that was released within five days (more than 50%), indicating that the use of CS NPs resulted in a significant increase in the wetting properties of the drug. A comparison of the two polyesters showed that PLGA is more suitable for the preparation of LFD patches, since the thin-films prepared showed better in-vitro release characteristics compared to PLLA. Finally, it should be noted that similar results were obtained for the ratio of 99.9/0.1 *w*/*w* polyester to LFD-CSnano (data not shown). 

## 4. Conclusions

In the present study, LFD-loaded CS NPs were successfully incorporated into PLLA and PLGA matrices for the preparation of drug-loaded thin-film skin patches. The CS NPs, prepared via an ionic gelation technique, were able to enhance LFD’s wetting characteristics successfully and, hence, improve its in-vitro dissolution properties when incorporated into the prepared polyester thin-films. Therefore, although further work is needed in order to evaluate the skin release characteristics, the present study shows that the combination of CS NPs with suitable polyesters is a promising solution for the preparation of drug-loaded skin patches utilizing drugs with poor wetting characteristics and low aqueous solubility.

## Figures and Tables

**Table 1:** LDF-loaded thin-film patches prepared via spin-coating technique.

Code	PLLA * (%wt.)	PLGA ^#^ (%wt.)		LFD (%wt.)
		75/25	50/50	
S1	95.0	-	-	5.0
S2	90.0	-	-	10.0
S3	80.0	-	-	20.0
S4	-	95.0	-	5.0
S5	-	90.0	-	10.0
S6	-	80.0	-	20.0
S7	-	-	95.0	5.0
S8	-	-	90.0	10.0
S9	-	-	80.0	20.0

* PLLA: poly(l-lactic acid); ^#^ PLGA: poly(lactic-co-glycolic acid).

**Table 2:** Drug loading and EE values of the prepared LFD-loaded polyester patches.

Sample-ID	Drug Loading (%)	*p*-Value *	EE (%)	*p*-Value *
PLLA-LFD (95/5 *w*/*w*)	4.98 ± 0.14	-	99.6 ± 0.31	-
PLLA-LFD (90/10 *w*/*w*)	9.35 ± 0.27	-	93.5 ± 0.82	-
PLLA-LFD (80/20 *w*/*w*)	19.20 ± 0.36	-	96.0 ± 1.06	-
PLGA 75/25-LFD (95/5 *w*/*w*)	4.54 ± 0.12	>0.05	90.8 ± 0.52	<0.05
PLGA 75/25-LFD (90/10 *w*/*w*)	8.36 ± 0.24	<0.05	83.6 ± 1.12	<0.05
PLGA 75/25-LFD (80/20 *w*/*w*)	18.33 ± 0.38	>0.05	91.7 ± 1.29	<0.05
PLGA 50/50-LFD (95/5 *w*/*w*)	3.33 ± 0.15	<0.05	66.6 ± 0.94	<0.05
PLGA 50/50-LFD (90/10 *w*/*w*)	7.39 ± 0.26	<0.05	73.9 ± 1.23	<0.05
PLGA 50/50-LFD (80/20 *w*/*w*)	17.23 ± 0.35	<0.05	86.2 ± 1.58	<0.05

* Significant values relative to the respective PLLA-LFD samples.

## Data Availability

Data are contained within the article.
